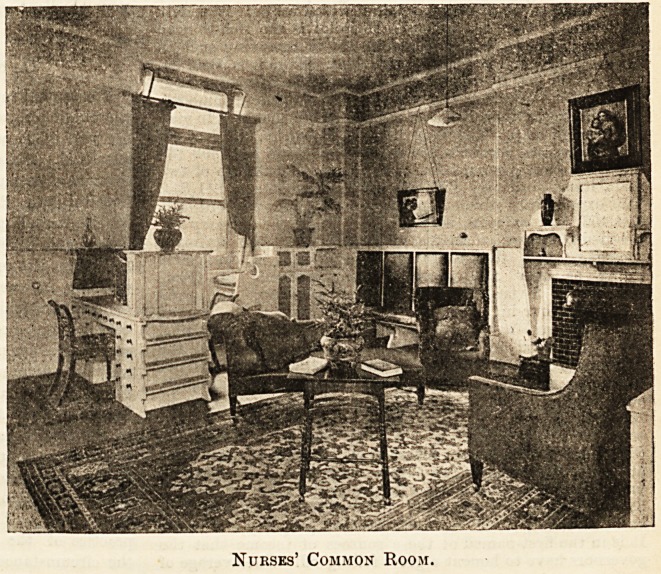# The Royal London Ophthalmic Hospital (Moorfields)

**Published:** 1900-04-28

**Authors:** 


### The Institutional Workshop.

### THE ROYAL LONDON OPHTHALMIC HOSPITAL (MOORFIELDS).

### THE NURSES' QUARTERS.

Visitors to the splendid new building in the City Road, which has taken the place of the ancient and inconvenient structure so well known to Londoners as the "Moorfields Hospital," close to Liverpool Street Stations, cannot have failed to be impressed by the excellence of the accommodation provided for the nursing staff. That every detail of the arrangements had been carefully thought out by those who knew exactly what was necessary and what was not is evident to any close observer. We have seen nurses' rooms of late years which have erred on the side of too much rather than too little luxury, but in this instance the happy mean seems to have been reached. Practical convenience and comfort reigns, but it is comfort and convenience of a reasonable and simple kind.

Nurses' Bedroom.

It is now some years since it was urged in The Hospital that for nurses' rooms, where every inch of space is of consequence, and bulky furniture is out of place, fixed fittings, adapted to the shape and size of each room, would be found to solve many difficulties. This plan has been well carried out at the Royal London Ophthalmic Hospital.

To take the bedrooms first, as illustrated by the accompanying photograph, notice (from a feminine point of view) the delightful possibilities in the way of cupboards, shelves, and drawers of all sizes which this method of having the furniture all made in one, so to speak, provides. There are cupboards for boots and bottles, and a special one for hats over the hanging wardrobe; small drawers provide space for toilet etceteras, and long ones for more bulky articles. The rooms are warmed by hot coils, near which is placed the rail for towels. The toilet glass is specially shaped so as to obstruct the light from the window as little as possible; the amplitude of the long glass, too, should make it inexcusable for any nurse to issue untidy from her bedroom. There are shelves for books, rails for pictures, and plenty of room for the trifles which all nurses like to gather round them wherever it is possible to do so. The washstand is fitted with marble slab and tiled back. The floors are covered with linoleum, and made pretty with a specially-designed strip of green carpet, matching in colour the dainty bedspreads. Polished pine being used for the fittings, the general effect is clean and bright, and it would be hard to find more thoroughly cosy little sanctums than those of which the "Moorfields" nurses are now possessed.

The second photograph shows the nurses' common room, a large well-lighted room, attractively fitted with white painted panelling and woodwork, with cosy

Nurses' Common Room.

corners and window-seats arranged to command a view of the busy road beneath, tiled hearth, polished and brightly carpeted floor, and restful chairs in plenty. Truly a pleasant "off duty" retreat. The writing-table, full of drawers and shelves, is cunningly designed, and along one wall stands a really fine specimen of a last century bookcase of mahogany, brought from the old hospital, a genuine treasure of its kind.

The late matron of the hospital, Miss Ada Robinson, spent much time and thought in making her nurses' quarters as perfect as possible, and certainly succeeded. The furnishing and fittings were admirably carried out by Messrs. Hampton, Pall Mall, by whose kind permission we reproduce the above photographs.

## Figures and Tables

**Figure f1:**
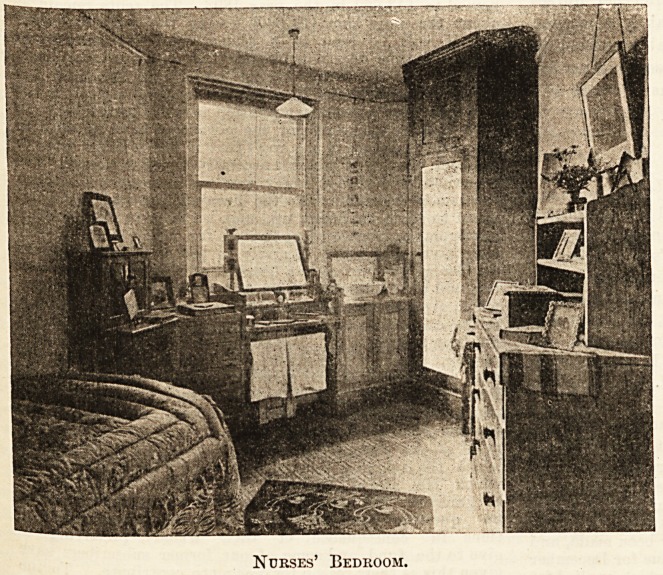


**Figure f2:**